# Enhanced nuclear information fusion and visual transformer for pathological breast cancer image classification

**DOI:** 10.1038/s41598-025-04344-2

**Published:** 2025-06-03

**Authors:** Qinyi Zhang, Honglei Gao, Wenhao Li, Zhipeng Xu, Ting Ouyang, Zongyun Gu

**Affiliations:** 1 College of Medical Information Engineering, Anhui University of Chinese Medicine, Hefei, China; 2 State Key Laboratory of Lunar and Planetary Sciences, Macau University of Science and Technology, Macau, China

**Keywords:** Pathological breast cancer image, Classification, Segmentation, Enhanced nuclear information fusion, Visual transformer

## Abstract

Breast cancer poses a significant threat to women’s health. Early diagnosis using pathological images is crucial for effective treatment planning. However, the low resolution of pathological images poses significant challenges for the extraction of valid information, while their high complexity greatly increases the difficulty of image analysis. To address these challenges, this paper introduces an innovative classification method for breast cancer histopathological images, combining enhanced nuclear information with an Enhanced Vision Transformer (EVT) model using wavelet position embedding. The quintessence of the proposed method resides in its capacity to efficiently extract both biological and foundational image features from pathological images. This is accomplished by initially enhancing nuclear information through the application of segmentation models and sophisticated image processing techniques. Subsequently, wavelet positional embedding within the EVT model is leveraged to precisely capture key information embedded within the images. Experimental outcomes have demonstrated that our method attains an accuracy rate of 94.61% and an AUC value of 99.07% on the BreaKHis dataset, significantly outperforming other baseline network models in terms of classification efficacy. Furthermore, through visual representation, this study underscores the significance of nuclear information enhancement and wavelet position transformation in the EVT model, thereby further confirming the effectiveness and effectiveness of the method we proposed.

## Introduction

Breast cancer is the most prevalent cancer among women globally, with significant incidence and mortality rates that pose a substantial threat to women’s health. Statistics indicate that annually, approximately 2.3 million new breast cancer cases are reported worldwide, with a slow yet consistent upward trend observed over the past 50 years^1^. In contemporary breast cancer management, precise diagnostic stratification and individualized treatment survival prediction are pivotal. However, this process largely hinges on the manual interpretation of pathological slides, which is not only time-consuming but also exhibits considerable observer-to-observer variability^2^.

The advent of microscopic image classification technology offers a new pathway to enhance the efficiency of breast cancer diagnosis. In recent years, the research significance of artificial intelligence (AI)^3^ in the medical field has become increasingly evident, with deep learning^4^ has been widely applied because of the diversity of its network structure. Among these, the convolutional neural network (CNN) has played an important role in medical image analysis, particularly in detection, classification, and segmentation tasks^5,6^. For instance, Jabeen et al. proposed to significantly improve the classification accuracy of breast lesions in ultrasound images by combining EfficientNet and ResNet deep network approaches using interpretable artificial intelligence techniques^7^. Nevertheless, the CNN’s local receptive field restricts its ability to directly capture long-range pixel relationships. Drawing inspiration from the success of a Transformer in natural language processing (NLP), Dosovitskiy et al.^8^ introduced the Vision Transformer (ViT), which possesses a crucial capability to establish long-term dependencies within the medical image receptive field^9^. However, the downsampling operation in ViT may lead to the propagation of erroneous information, thereby inducing aliasing effects^10^. To address this issue, Ding et al.^11^ proposed an enhanced wavelet position embedding vision transformer and applied it to the study of pathological image classification.

**Fig. 1:**
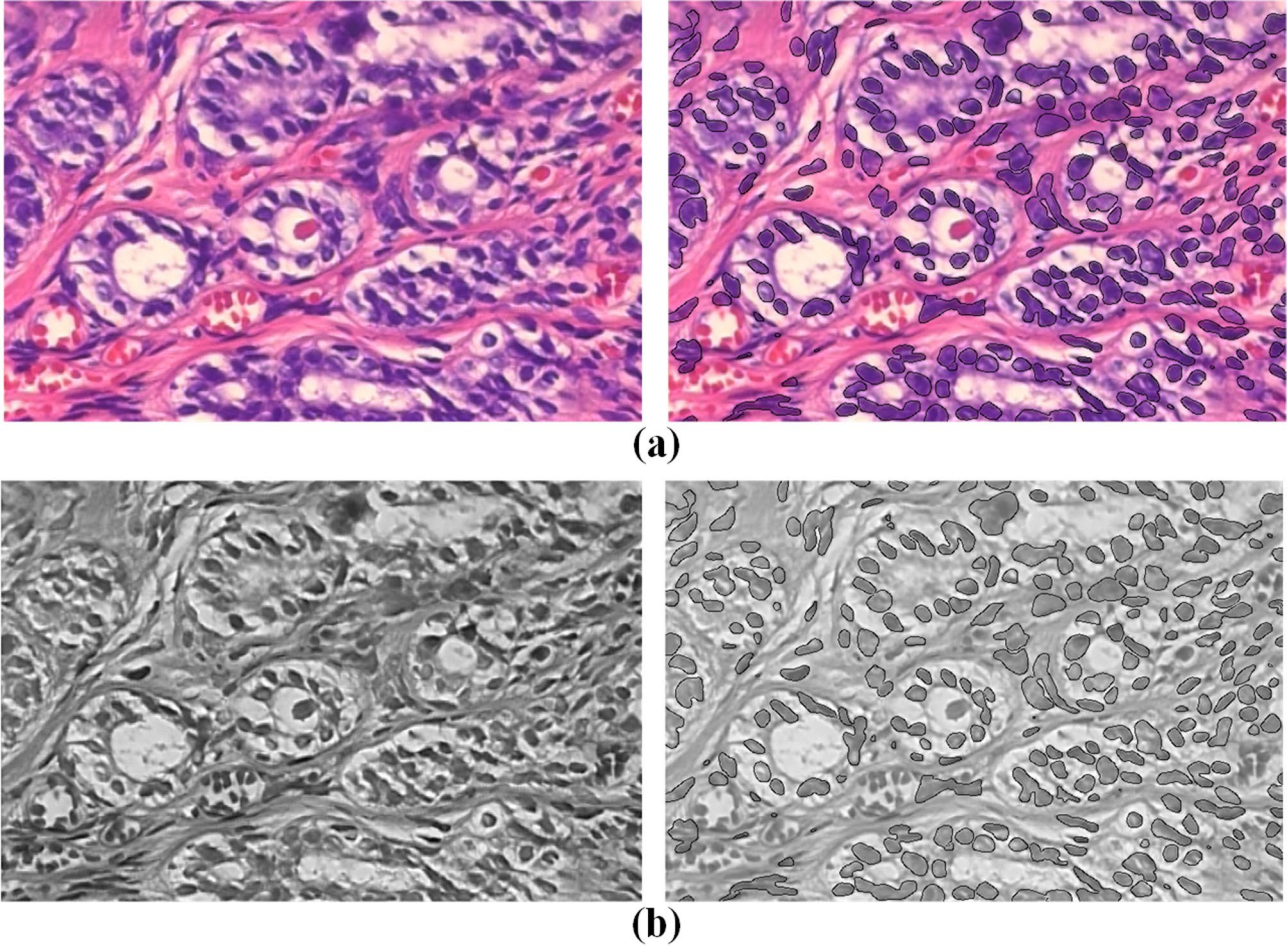
Shows the comparison of details, with raw data on the left and enhanced data on the right. (**a**) Differences before and after image enhancement. (**b**) The difference of channel 3 after normalization before and after image enhancement.

With the progression of AI, research on digital pathological images is surging, and high-resolution data has emerged as the cornerstone of such investigations. In practical applications, the processing of low-resolution images is equally important. Feature extraction^12^, a vital step in deriving useful information from images, reducing data dimensionality, and enhancing model generalization capability, holds particular significance in pathological image analysis. Primary strategies encompass learning general image features and detecting important biological components, such as the nucleus, while quantitatively characterizing their abundance and spatial organization^13^. As foremost biological element in the tissue section, the precise definition of the nucleus is indispensable for enhancing diagnostic accuracy. Compared with the machine learning algorithm, the segmentation model employed for nucleus extraction can delineate nuclear boundary with greater stability and precision, mitigating the impact of image noise, as illustrated in Fig. 1.

The purpose of this study is to achieve automatic classification of pathological images utilizing deep learning technology, thereby enhancing diagnostic efficiency and accuracy. Enhanced nuclear data furnish biometric features for the network, and we introduce the enhanced wavelet position embedded vision transformer (EVT_wps) to process these image features, aiming to attain precise classification of breast cancer pathological images. The key contributions of this study are as follows:

- A novel automatic classification framework for pathological images in low-resolution scenarios is constructed, significantly improving the classification accuracy of breast cancer images.
- An innovative nuclear information enhancement strategy is proposed and integrated into the baseline model, effectively enhancing the classification performance by highlighting nuclear features.
- EVT technology based on wavelet position embedding is introduced for breast cancer image classification, and its superiority in combination with nuclear information enhancement for processing complex pathology images is experimentally verified.

## Related works

### Analysis of pathological images of breast cancer

Breast cancer is an epithelial malignancy that originates from the terminal ductal lobular unit of the breast. Its histological morphology is exceptionally complex and can be broadly categorized into two types: non-invasive cancer and invasive cancer. Among these, invasive ductal carcinoma is the most prevalent form of breast cancer, accounting for up to 70% of cases. For a considerable period, changes in nuclear morphology have served as a crucial indicator in cancer pathology^14^. In breast cancer cells, nuclei typically exhibit enlargement and significant alterations in shape, which are observed throughout the clinical progression of the disease^15,16^.

In the study of breast cancer pathological images, there exists a close correlation between morphology features and underlying molecular characteristics^17^. This observation holds significant implications for the application of AI technology in breast cancer research. Specifically, the underlying molecular changes and biological properties can be inferred from the image features. The success of this inference process heavily relies on the quality of the image, particularly their resolution^18^. High-resolution images enable clear visualization of the microstructure and characteristics of breast cancer tissue, including nucleus shape, cellular arrangement, and blood vessel distribution, which are vital for accurate analysis and inference by AI models^5^.

However, in practical applications, obtaining images of sufficient resolution can be challenging due to various factors, such as technical limitations, sample processing issues, or storage conditions. Under such circumstances, the significance of low-resolution images becomes particularly evident. Although low-resolution images may not provide the same level of detail as a high-resolution one, they still contain valuable information^19^. Extracting key features from low-resolution images, such as specific textures, colors, shapes, etc., is equally crucial for the diagnosis and classification of breast cancer. Consequently, effectively utilizing image information in the case of low resolution has emerged as a critical challenge that needs to be addressed.

### Deep learning of breast cancer

The rapid advancement of deep learning technology has enabled the practical application of computer-aided histopathological image classification in cancer diagnosis and prognosis prediction, attracting significant attention from researchers. As is well known, CNNs have demonstrated remarkable performance across various computer vision tasks^20^.

Digital pathology advances have also produced valuable datasets for precision medicine and disease research. Fatima et al. proposed a method for segmentation and classification of breast lesions based on U-Net significance estimation and interpretable residual convolutional neural networks^21^.. Recent work by Ortega-Ruíz et al. introduced DRD-UNet, an enhanced UNet variant for precise breast cancer segmentation in pathological images^22^. A new contrast enhancement technique was introduced to improve the quality of the original image. Ding et al. contributed a large-scale SHOW dataset, employing a standardized workflow and a ready-made image generator and kernel annotator^23^. Their experimental validation confirmed that ResUnet exhibits high adaptability on the SHOW dataset, achieving commendable segmentation results.

Spanhol et al. introduced the BreaKHis dataset, focusing specifically on pathological images of breast cancer^24^. They investigated the performance of 24 combinations of four classifiers in classification tasks, integrating support vector machine (SVM) with artificially designed texture features, such as adjacency threshold statistics. Their results indicated an accuracy range of 80% to 85% in distinguishing between benign and malignant breast tumors, establish in an important benchmark for the field.

On this basis, Seo et al. developed a scalable multi-instance support vector machine (SVM) framework for breast cancer detection on the BreaKHis dataset, achieving robust performance in histopathological image analysis^25^. Xiao et al. proposed a convolutional neural network (CNN) framework for breast cancer classification using cytopathology images, demonstrating its effectiveness in automated cancer diagnosis^26^. Simonyan et al. proposed a CNN-based approach for histopathological breast cancer classification, achieving an accuracy of 91.37% on the BreaKHis dataset^27^. Their results demonstrate the potential of deep learning in improving diagnostic precision for breast cancer.

Despite the widespread application of CNNs in breast cancer pathological image classification and the repeated validation of their effectiveness using the BreaKHis dataset, there remains a need for further accuracy improvements. A comprehensive performance assessment should incorporate multiple complementary metrics including the F1-score, Youden’s index, and the area under the ROC curve (AUC) to provide a more rigorous evaluation of model robustness^28^.

### Transformer of pathological images

In recent years, Transformers^29^ have become a cutting-edge technology in the field of deep learning, capable of achieving robust results in a variety of computer vision tasks. Atabansi et al.^30^ pointed out that CNNs have limitations in modeling telematic and spatial dependencies, posing numerous challenges. In contrast, the Transformer architecture has demonstrated excellent performance in pathological image processing. In the field of histopathological imaging^31^, Transformer methods have been widely used in multiple aspects such as image segmentation^32^, classification^33,34^, detection^35,36^, representation^37,38^, cross-modal retrieval^39,40^, image generation^41,42^, survival analysis^43,44^, and survival prediction^45,46^.

Sriwastawa and Arul Jothi conducted a comparative study of vision transformers for breast cancer histopathology classification, reporting a top accuracy of 92.12% in their experiments^47^. Spangenberg et al.^48^ conducted a comprehensive benchmark test on five commonly used pathology datasets, comparing the latest Vision Transformer (ViT), CNNs, and their hybrid models, to evaluate the effectiveness of different classification models in histopathology image classification. They tried to incorporate convolution operations into the ViT architecture and explored two ways to combine them: one to introduce convolution in the initial phase, and the other to embed the convolution layer into each Transformer block. The experimental results demonstrate that regardless of the adopted combination strategy, the fusion of CNNs and ViT exhibits significant advantages. However, as pointed out by Qian et al., convolution blocks in the feature extraction process are prone to generating discontinuous information, which may cause confusion once it flows into the Transformer module^49^.

To address this issue, inspired by the work of Ding^11^, this paper adopts an innovative solution, the EVT incorporating wavelet position embedding technology. Wavelet position embedding is not only excels representing smooth and low-frequency components of feature maps but also in accurately capturing fine details and high-frequency parts. By incorporating wavelet position embedding, the EVT effectively addresses the aliasing phenomenon, demonstrating superior performance in processing pathological images and efficiently managing feature information.

## Methods

### Overview

**Fig. 2:**
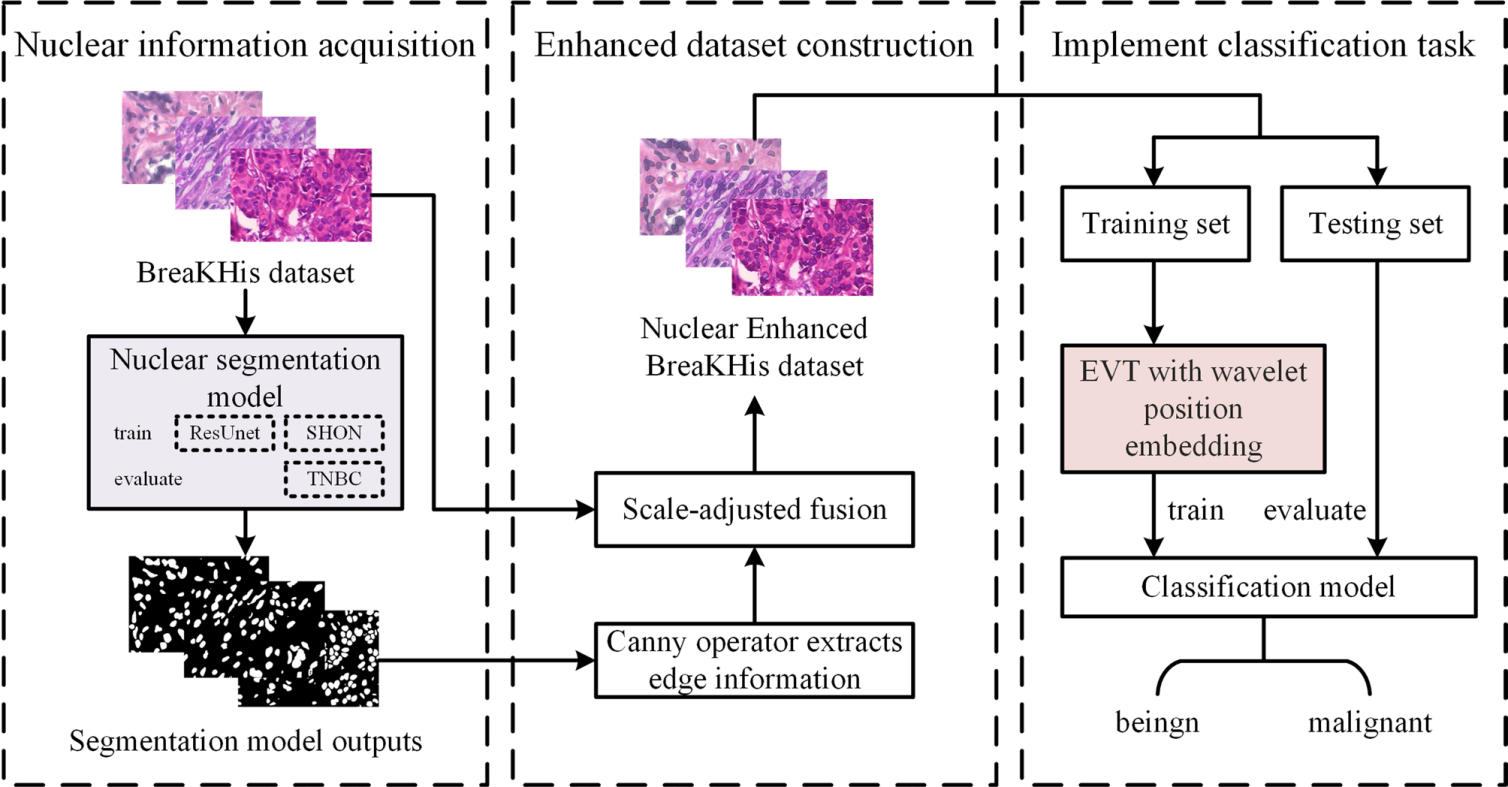
The overall flow of the method. The framework consists of three main stages: (1) Nuclear information acquisition, where the BreakHis dataset is processed by a segmentation model (2) Enhanced dataset construction, where the segmented nuclei are refined via scale-adjusted fusion and (3) Classification task implementation, where the enhanced data is.

The automatic classification scheme proposed in this research is depicted in Fig. 2, which specifically covers the following three core steps:

Firstly, the edge information of the nucleus is extracted. The nuclear segmentation model was trained using the SHOW data set and ResUnet architecture. This trained model is then employed to successfully extract the edge information of nuclei from pathological images.

Secondly, an enhanced breast cancer pathology image dataset is generated. The classification data set is input into the segmentation model, and the segmentation image is fused with the original image through data processing technology, to construct a data set with significantly enhanced nuclear information.

Lastly, the classification task is executed. Leveraging the enhanced dataset obtained in Step 2, the EVT embedded with wavelet position is integrated to train.

### Nuclear information enhancing

Given that diagnostic pathologists diagnose malignant tumors based on the morphological abnormalities of nuclei^50^, it is plausible that incorporating enhanced nuclei information into the training data could enhance the model’s ability to recognize and interpret the morphological features of nuclei, potentially improving its performance in screening for malignant tumors.

Consequently, this study aimed to label the nuclei in pathological images prior to inputting them into the classification network, thereby enriching the nuclear information within the images. We trained a ResUnet model on the SNOW dataset. During training, domain discrepancies between synthetic and real-world data were mitigated through data augmentation techniques such as rotation, flipping, and intensity variation. A hybrid BCE-Dice loss function was employed to ensure robust model convergence, while the StepLR scheduler was utilized to prevent the model from becoming trapped in local optima.

**Fig. 3:**
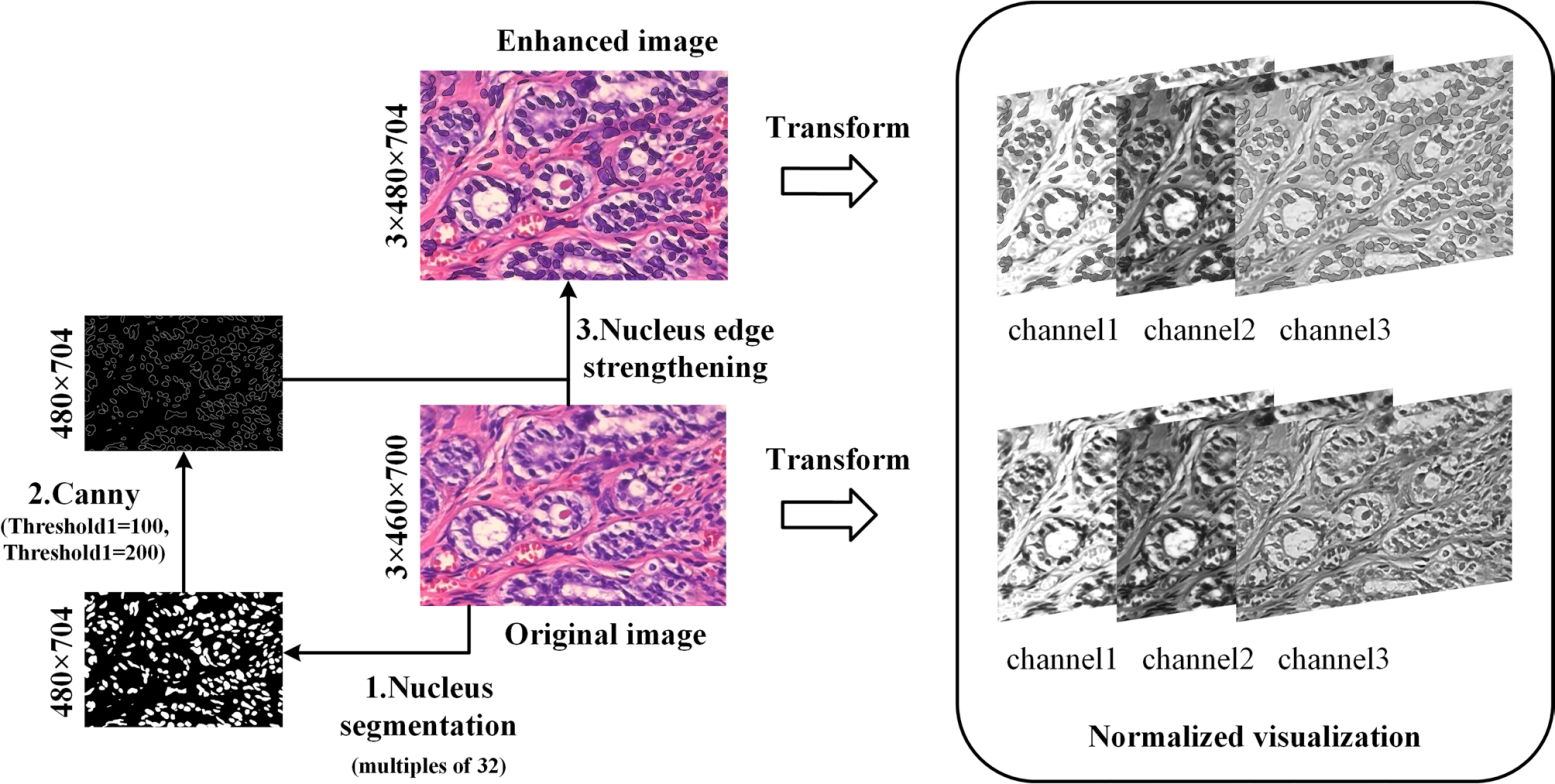
Schematic diagram of nuclear fusion method. The pipeline processes histopathology images through nucleus segmentation, canny edge detection and edge strengthening.

To enhance nuclear information for subsequent classification, this study implemented a preprocessing pipeline involving precise nuclear segmentation followed by edge augmentation. The workflow (Fig. 3) comprises four key stages:

First, we trained a ResUnet segmentation model on the SNOW dataset, employing rotation, flipping, and intensity variation augmentations to bridge the synthetic-to-real domain gap. The model’s convergence was stabilized using a hybrid BCE-Dice loss function with StepLR scheduling to avoid local optima.

For each input image, we initially resized it to a 32-divisible dimension before feeding it to the trained segmentation model. The nuclear masks are the segmentation results obtained using the ResUnet model. And the nuclear masks, rather than the original images, served as the basis for Canny edge detection. The Canny algorithm extracts edges by retaining pixels with gradient intensities between 100–200. This critical distinction ensures morphological boundaries are extracted exclusively from segmented nuclei regions. The resulting contours were then multiplicatively fused with corresponding original images to create enhanced representations that emphasize diagnostically relevant nuclear features.

Finally, the augmented images were normalized using the standard ImageNet parameters ($mean=[0.485, 0.456, 0.406]$, $std=[0.229, 0.224, 0.225]$) and converted to 3-channel tensors for classification model training and validation.

Upon examining the third channel of the transformed enhanced image, it becomes evident that the nuclear boundary is prominently highlighted, while the surrounding tissue appears relatively subdued (Fig. 1). Canny edges were multiplicatively fused with the original image to enhance nuclear morphology. This step allowed the EVT model to place relatively greater emphasis on diagnostically relevant features. This demonstrates that the method effectively retains the surrounding tissue information while emphasizing the nuclear boundary in the image, which holds significance promise for in-depth analysis of the nucleus and its surrounding environment.

### An EVT with wavelet position embedding

**Fig. 4:**
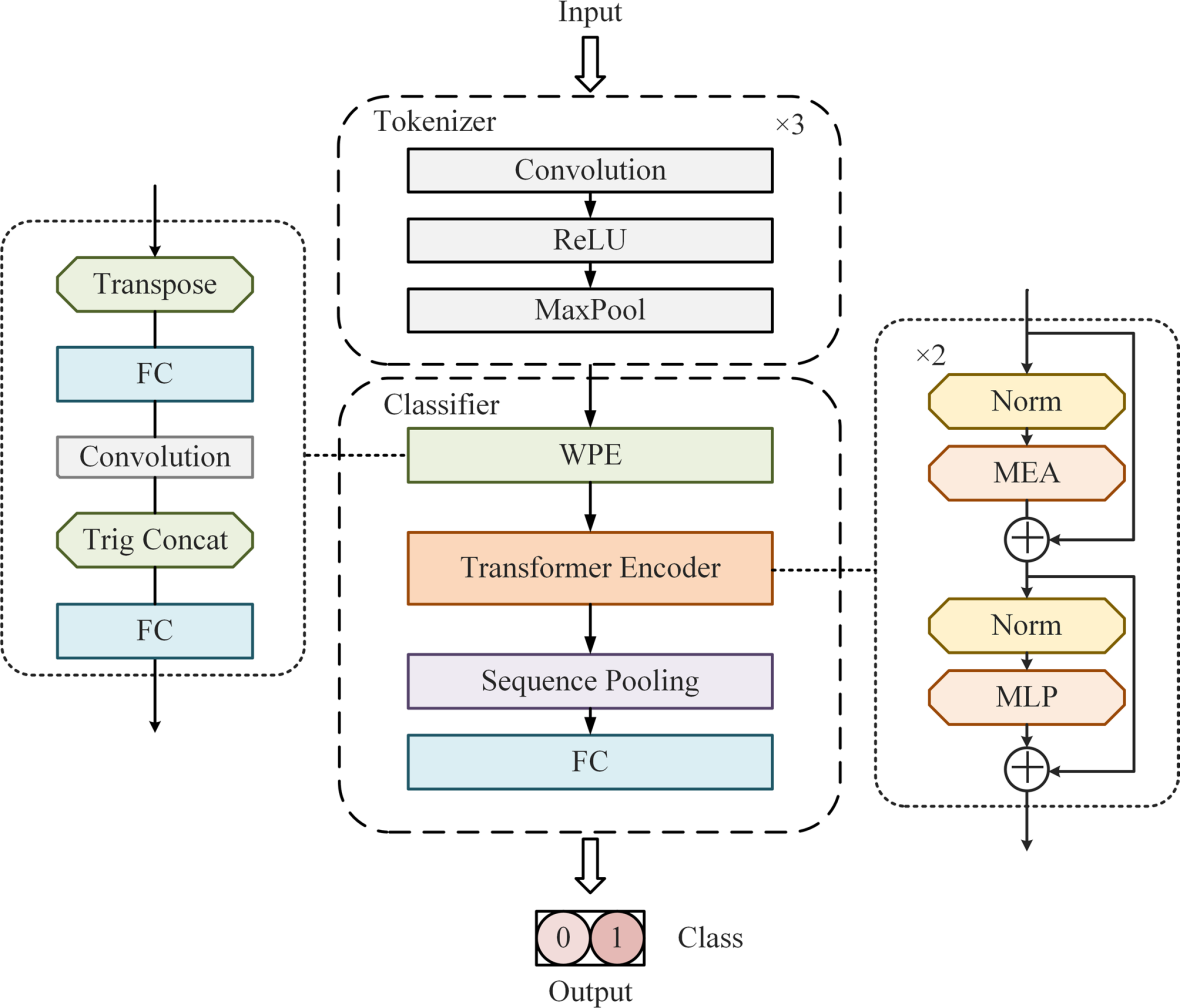
EVT structure. The network processes input through tokenization blocks (convolution/ReLU/pooling), parallel branches (transpose/FC/attention), and a classifier with wavelet position embedding, ultimately outputting binary classification results.

The EVT_wpe^11^ is an end-to-end trainable architecture comprising a marker, a wavelet position embedding module, a transformer encoder, a sequence pooling layer, and a full convolution (FC) classifier module. The core innovation of this architecture lies in the introduction of wavelet transform for position embedding tasks, aiming to reduce aliasing effects during downsampling. Unlike fixed sinusoidal embeddings, WPE adapts to image patches’ spectral properties via discrete wavelet transform (DWT), minimizing aliasing artifacts during tokenization (Fig. 4). This is critical for pathological images where nuclear boundaries are high-frequency signals.

In this study, we applied EVT_wpe architecture to train a breast cancer pathological image classification model, using nuclear-enhanced image data to achieve image feature extraction and classification. As depicted in Fig. 4, the tokenizer consists of three convolution blocks, each comprising a convolution layer, an activation layer, and a MaxPool layer. One of the wavelets transform technique (WPE) module processes can be expressed as follows: First, the input data is adjusted. Next, a proportional value is obtained by linear computation, then the data is convolved. A new value is computed by a trigonometric function, and then these values are combined. Finally, the processed results are merged with the original data. This process combines operations such as linear computation, convolution, and trigonometry, which are combined with augmented kernel data to extract and update features in the data. In addition, The Transformer Encoder module primarily consists of multiple multi-head attention (MHA) modules and MLP (multi-layer perceptron) modules.

The workflow of the model is as follows: Initially, the input image is processed by the tokenizer module to obtain the low-dimensional representation of the histopathological image. Subsequently, the feature-mapped WPE serves as the input to the transformer, enabling the extraction of long-range information from image patches. The Sequence information generated by the transformer encoder is then pooled using sequence pooling layer. Finally, the FC classifier output the classification results.

## Experimental results and discussion

### Datasets

In this work, we utilized three distinct breast cancer pathological image datasets for the training and classification experiments of the nuclear segmentation model: SNOW^23^, TNBC^13^, and BreaKHis^24^, as detailed in Table 1. both SNOW and TNBC datasets have an image resolution of 512 × 512 pixels. The SNOW dataset was randomly partitioned into a training set and a verification set in a 95:5 ration. The TNBC dataset was used to do the testing and evaluation of the segmentation model, which is a subtype of breast cancer with unique imaging characteristics and complex pathological and imaging manifestations that make it ideal for validating the effectiveness of the segmentation algorithms and the potential for clinical applications^51^. On the other hand, the BreaKHis dataset comprises benign and malignant images of breast cancer pathology, with the objective of automatically classifying these images into two categories. Specifically, within the BreaKHis dataset, it was randomly divided into training, validation, and test sets in a 7:2:1 ratio. The test set was kept consistent for the test-contrast phase, containing 248 benign and 531 malignant samples.

**Table 1:** Data set description.

Dataset	Characteristic	Sample size	Experimental use
SHOW	A large-scale dataset of Synthetic pathology images, combined with annotations for nuclear semantic segmentation, called the Synthetic Nuclei and annOtation Wizard	20,000	Training of nuclear segmentation models
TNBC	From 11 patients with Triple Negative Breast Cancer, the dataset represents variability between patients with the same cancer type, with numerous cells documented in detail in the dataset	4022	Test and evaluate segmentation models
BreaKHis	From 82 patients, including benign and malignant images	7783 5304 (malignant samples) 2479 (benign samples)	Training and validation of classification models

### Evaluation metrics

We evaluated the performance of the nuclear segmentation model using the Dice score, Jaccard score, and Hausdorff distance. The Dice score, presented in Formula 1, is used to measure the similarity between two samples, calculated by doubling the intersection inter to increase its weight and dividing by the sum of the predicted region, target region, and smooth terms. The Jaccard score, shown in Formula 2, reflects the degree of overlap between the predicted and real region. The Hausdorff distance, as described in Formula 3, is $A$ distance parameter between two subsets in a metric space, for each point in a point set a, its maximum distance to the nearest point in another point set $B$ where $d(a,b)$ represents the distance between points $a$ and $b$.

1 $$\begin{array}{c}dice=\frac{2*inter+smooth}{su{m}_{pred}+su{m}_{targ}+smooth}\end{array}$$

2 $$\begin{array}{c}jaccard=\frac{inter+smooth}{su{m}_{pred}+su{m}_{targ}-inter+smooth}\end{array}$$

In the above two equations, the prediction result tensor $pred$ matches the shape of the comment label tensor $targ$, where $\text{inter}={\text{sum}}_{\text{pred}\times \text{trag}}$ is the global sum obtained after calculating the multiplication $pred\times targ$, represents the sum of the product of all elements in the two tensors and represents the intersection between the prediction region and the target region. The smooth value is set to 1/1e32 to avoid division by zero. $su{m}_{pred}$ and $su{m}_{targ}$ represent the sum of the tensors $pred$ and $targ$, respectively.

3 $$\begin{array}{c}\hbox{Hausdorff}=\text{H}\left(\text{A},\text{B}\right)=\underset{\text{a}\in \text{A}}{\text{max}}\left\{\underset{\text{b}\in \text{B}}{\text{min}}\left\{d\left(\text{a},\text{b}\right)\right\}\right\}\end{array}$$

To evaluate the classification effect on the BreaKHis dataset, this study adopted a few technical indicators, including Accuracy, Youden index, Precision of various categories, Recall, and F1 scores, to comprehensively evaluate the performance of the classification model in the screening of breast cancer pathological images. In addition, an in-depth comparative analysis of data augmentation and wavelet position transforms was performed in EVT by calculating AUC and Kappa coefficients.

In the test sample, accuracy is defined as the proportion of samples that the model correctly classifies. The F1 score is the harmonic average of the accuracy rate and the recall rate, calculated as shown in Formula 4. In this study, there may be inference errors in the model, such as false positives or false negatives in a broad sense, which can be considered diagnostic errors or incorrect identification of positive samples. Therefore, the Youden index, as a comprehensive evaluation index combining Sensitivity and Specificity, was suitable for evaluating the performance of a specificity model, and its calculation was included in Formula 5.

4 $$\begin{array}{c}{F}_{1}=2\times \frac{Precision\times Recall}{Precision+Recall}\end{array}$$

5 $$\begin{array}{c}Youden=Sensitivity+Specificity-1\end{array}$$

The AUC is a quantitative representation of the area under the ROC curve used to evaluate the overall performance of a classification model. The horizontal coordinate of the ROC curve represents the false positive rate, that is, the probability that benign is incorrectly predicted as malignant. The ordinate represents the true positive rate, which is the likelihood that malignant is correctly predicted as malignant. Kappa is a measure of classification accuracy and is often used for consistency testing, which is calculated through the confusion matrix, as shown in Formula 6. ${p}_{0}$ has the same meaning as accuracy, and ${p}_{e}$ is the sum of the product of actual and predicted quantities corresponding to all categories divided by the square of the total number of samples.

6 $$\begin{array}{c}kappa=\frac{{p}_{0}-{p}_{e}}{1-{p}_{e}}\end{array}$$

### Experimental details

In the training process of the nuclear segmentation model, we utilized the Adam optimizer in conjunction with a StepLR scheduler to adjust the learning rate based on the iteration step length. Specifically, the learning rate was multiplied by 0.1 every 30 iterations. Additionally, we set the random seed to 21, 42, 84 (to ensure reproducibility), the number of epochs to 100, and employed a loss function that combine binary cross-entropy loss (BCE) and Dice scores in a 1:1 ratio.

For the comparison experiment, we selected typical methods with proven performance on ImageNet as the control group, including VGG16, AlexNet, GoogleNet, ResNet34, MobileNetv2, and ViTb/16. To enhance computational efficiency, we resize the image to 224 × 224, noting that this may be result in pixel loss. Select the best model weights for inference on the test set and observe the convergence trend.

Regarding the optimization strategy, the Adam optimizer was for the first five models, whereas the stochastic gradient descent (SGD) method was employed for the ViTb/16 model. The ViT-B/16 model has a very large parameter space and SGD may be more effective in this case to avoid falling into local minima. To clarify, epochs were consistently set to 100 for all models. It is worth mentioning that the EVT_wpe model was also trained using the Adam optimizer with the hyperparameters listed in Table 2 (100 epochs, 5 warm-up steps, batch size of 2, initial learning rate of 0.001, and weight decay of 0.0001).

**Table 2:** Common hyperparameter configurations for EVT model training.

Parameter	Description	Value
Epochs	Total training rounds	100
Warmup	Warmup steps	5
Batch size	Samples per training iteration	2
lr	Initial learning rate	0.001
Weight decay	L2 regularization factor	1e-4

It is worth noting that during the inference phase of the ablation experiments, we employed CPU-based computation and disabled all stochastic operations (including but not limited to random initialization and random sampling). Additionally, we ensured batch consistency by fixing the batch size to 1 and performing sequential inference in a predetermined order. These measures effectively mitigate the influence of random factors on the inference results, thereby guaranteeing the reproducibility of the findings.

### Effect of trained nuclear segmentation model

Before extracting nuclei from pathological images of breast cancer, it is essential to construct a segmentation model suitable for pathological images. Inspired by the work of Kexin Ding^23^ et al., we utilized the SHOW dataset, which features a diverse set of breast cancer pathology images, in conjunction with the ResUnet network to train our segmentation model. This model was then validated on the TNBC^13^ dataset. The detailed description of relevant datasets are provided in Table 1.

The results of the evaluation are shown in Table 3, and it is worth noting that we did not use ImageNet as pre-training data in our work. Through observation and analysis of the data in the table, it can be found that the Dice of all models exceeds 0.75, which indicates that they all can segment the nucleus. Especially, when the seed of model weight saving is 42, the model has the best comprehensive performance in image segmentation.

**Table 3:** Results of the training stage of the segmentation model.

Model	Seed	Dice	Jaccard	Hausdorff
	21	0.78649	0.65848	7.30393
ResUnet	**42**	0.79294	0.66642	7.30142
	84	0.77985	0.65219	7.17857

Significant values are in [bold].

**Fig. 5:**
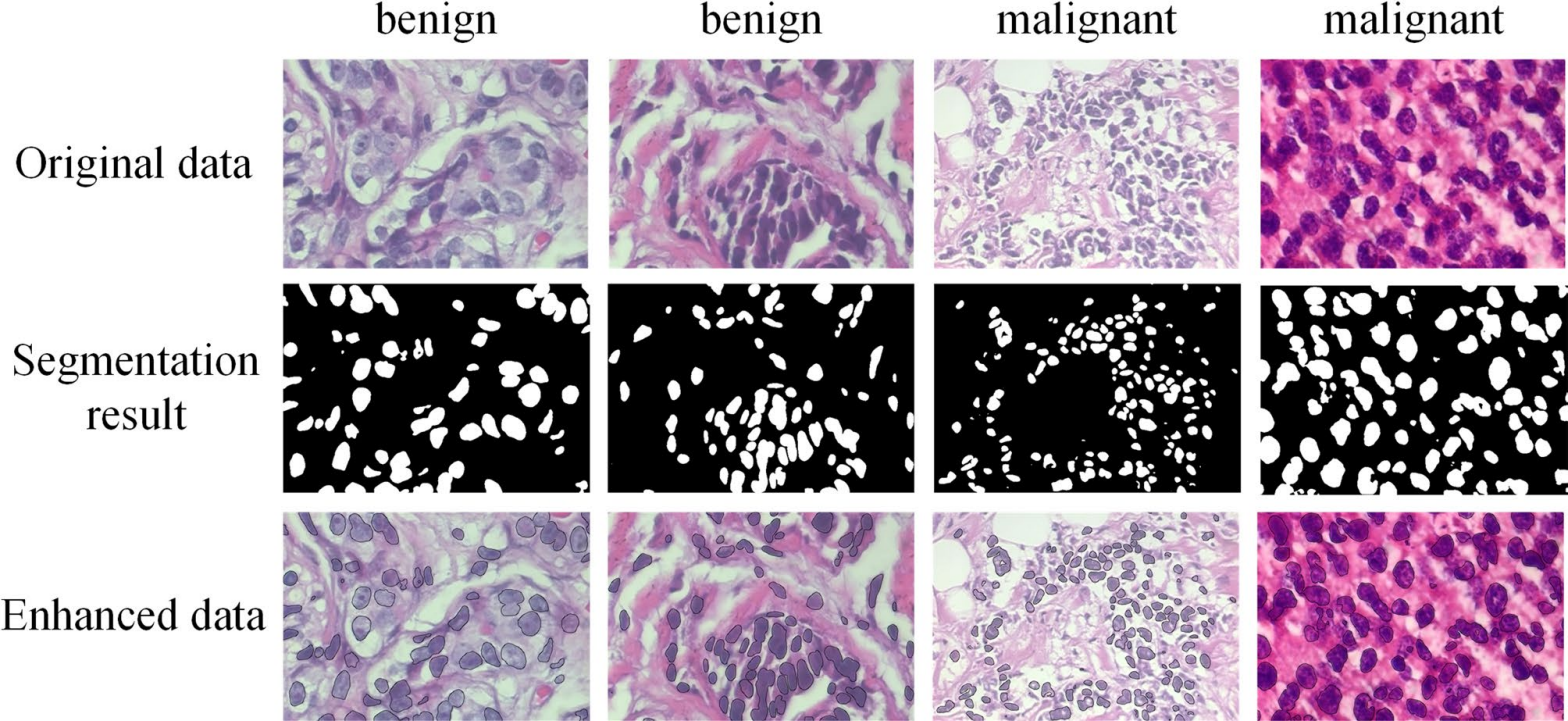
Comparison of original data, nucleus segmentation results, and enhanced data for benign and malignant cases.

Therefore, we selected the model with the seed of 42 for further image processing and randomly chose four images with widely distributed nuclei for display. The segmentation results of these selected images are presented in Fig. 5, which fully satisfies the requirement for improved nuclear segmentation in the images.

### The performance of the classification models

#### Comparison reference model

Aiming at the classification task of breast cancer pathological images, this paper conducted a detailed experimental analysis, compared the performance of various network structures, and summarized the results in Table 4. In view of the significant influence of network depth on model convergence speed, to fully harness the performance potential of each model, we introduced pre-training weights for GoogleNet, ResNet34, MobileNetV2 and ViTb/16, aiming to use the advantages of transfer learning to accelerate the model training process and improve the final classification performance.

**Table 4:** Summary table of the test performance of each model on the original data and enhanced data. If the accuracy, F1 score and Youden result are improved by more than 1% compared with the results of origin data, they are marked with ↑; otherwise, they are less than 1%, marked with ↓.

Model	Date Type	Acc	Class	Pre	Rec	F1	Youden
VGG16	*Original*	0.8930	*Benign*	0.847	0.81	0.828	0.742
			*Malignant*	0.913	0.932	0.922	
	*Enhanced*	0.9008	*Benign*	0.854	0.83	↑0.842	↑0.764
			*Malignant*	0.922	0.934	0.928	
AlexNet	*Original*	0.8892	*Benign*	0.821	0.834	0.827	0.749
			*Malignant*	0.922	0.915	0.918	
	*Enhanced*	0.8802	*Benign*	0.799	0.835	↓0.817	↓0.737
			*Malignant*	0.921	0.902	0.911	
GoogleNet	*Original*	0.9330	*Benign*	0.895	0.895	0.895	0.846
			*Malignant*	0.951	0.951	0.951	
	*Enhanced*	↑0.9472	*Benign*	0.908	0.927	↑0.917	↑**0.884**
			*Malignant*	0.966	0.957	↑0.961	
ResNet34	*Original*	0.8866	*Benign*	0.799	0.863	0.830	0.761
			*Malignant*	0.933	0.898	0.915	
	*Enhanced*	↑0.8995	*Benign*	0.866	0.81	0.837	↓0.751
			*Malignant*	0.914	0.941	↑0.927	
MobileNetv2	*Original*	0.9059	*Benign*	0.863	0.839	0.851	0.777
			*Malignant*	0.925	0.938	0.931	
	*Enhanced*	0.9021	*Benign*	0.857	0.829	0.843	↓0.765
			*Malignant*	0.922	0.936	0.929	
DenseNet	*Original*	0.9422	*Benign*	0.932	0.883	0.907	0.853
			*Malignant*	0.947	0.97	0.958	
	*Enhanced*	0.9448	*Benign*	0.96	0.863	0.909	0.846
			*Malignant*	0.939	0.983	0.960	
ViTb/16	*Original*	0.8299	*Benign*	0.796	0.629	0.703	0.553
			*Malignant*	0.841	0.924	0.881	
	*Enhanced*	0.8299	*Benign*	0.805	0.617	0.699	0.547
			*Malignant*	0.838	0.93	0.882	
**EVT_wpe**	*Original*	0.9461	*Benign*	0.929	0.899	0.914	0.867
			*Malignant*	0.954	0.968	0.961	
	*Enhanced*	**0.9487**	*Benign*	0.923	0.915	**0.919**	↑0.879
			*Malignant*	0.961	0.964	**0.962**	

Significant values are in [bold].

The BreaKHis dataset has significantly more malignant samples than benign samples, and this imbalance makes it difficult for the traditional accuracy (Acc) metric to fully reflect the model’s ability to classify the minority class (benign samples). Therefore, this study focuses on Recall (Rec), F1 Score and Youden Index, which are metrics that can more comprehensively measure the model’s performance when dealing with imbalanced data. Recall measures the model’s ability to recognise positive samples, the F1 score combines precision and recall, and the Youden index is used to assess the model’s balance and effectiveness in classification tasks. These metrics are particularly important when assessing the model’s ability to detect minority classes (benign samples).

As can be seen from the tabular data, the EVT_wpe model performs particularly outstandingly on the augmented dataset. For the malignant samples, the recall rate of EVT_wpe reaches 0.964, and the F1 score is 0.962, demonstrating its superior performance in handling the majority class (malignant samples). Meanwhile, for the minority class (benign samples), the recall rate of EVT_wpe is 0.915, and the F1 score is 0.919. The Youden index reaches 0.879, showing its ability to effectively identify minority class samples while maintaining a high recall rate for the majority class.

In comparison, other models perform slightly less well on the augmented dataset. For example, VGG16 has a recall rate of 0.934 and an F1 score of 0.928 for malignant samples on the augmented dataset, with a Youden index of 0.764. Although its performance is good, the Youden index is significantly lower than that of EVT_wpe. AlexNet and MobileNetv2 both show a slight decrease in various metrics on the augmented dataset and are the least suitable models for nuclear data augmentation in this study.

Compared with other models, GoogleNet has a recall rate of 0.957 and an F1 score of 0.961 for malignant samples on the augmented dataset, with a Youden index of 0.884. Although its performance is close to that of EVT_wpe, it falls slightly short in identifying the minority class, with a recall rate of 0.927 and an F1 score of 0.917. ResNet34 has a recall rate of 0.941 and an F1 score of 0.927 for malignant samples on the augmented dataset, with a Youden index of 0.751. It performs well on the majority class but still has room for improvement in identifying the minority class. DenseNet has a recall rate of 0.983 and an F1 score of 0.960 for malignant samples on the augmented dataset, with a Youden index of 0.846. Although it achieves a very high recall rate for the majority class, its recall rate for the minority class is only 0.863, with an F1 score of 0.909, indicating its deficiency in identifying the minority class. Lastly, ViTb/16 has mediocre performance on the majority class and significant shortcomings in identifying the minority class on both datasets, making it the least effective model overall.

Considering recall, F1 score and Youden index together, EVT_wpe performs well on the augmented dataset, especially when dealing with unbalanced data, and can effectively balance the ability to recognise the majority class (malignant samples) and the minority class (benign samples).

#### Ablation experiment

In the ablation experiments of this study, we have deeply explored the effects of nuclear information enhancement (nie) and wavelet position transform (wpe) modules on the performance of EVT models, aiming to reveal their key roles.

The experimental results are shown in Table 5, which verifies that the wpe module extracts the position information in the image by wavelet transform and enhances the model’s ability to capture the spatial structure features of the image. The experimental results show that after the introduction of the wpe module, the EVT + wpe model significantly outperforms the base EVT model in all indicators.

**Table 5:** Ablation experiment results showing the impact of nie and wpe modules on EVT model performance, including accuracy, precision, recall, F1 score, Youden index, and Kappa coefficient.

Model	Acc	Class	Pre	Rec	F1	Youden	Kappa
EVT	0.9345	*Benign*	0.899	0.895	0.897	0.848	0.8489
		*Malignant*	0.951	0.953	0.952		
EVT + wpe	0.9461	*Benign*	0.929	0.899	0.914	0.867	0.8747
		*Malignant*	0.954	0.968	0.961		
EVT + nie	0.9397	*Benign*	0.921	0.887	0.904	0.851	0.8597
		*Malignant*	0.948	0.964	0.956		
**EVT + wpe + nie**	**0.9487**	*Benign*	0.923	0.915	**0.919**	**0.879**	**0.8814**
		*Malignant*	0.961	0.964	**0.962**		

Significant values are in [bold].

**Fig. 6:**
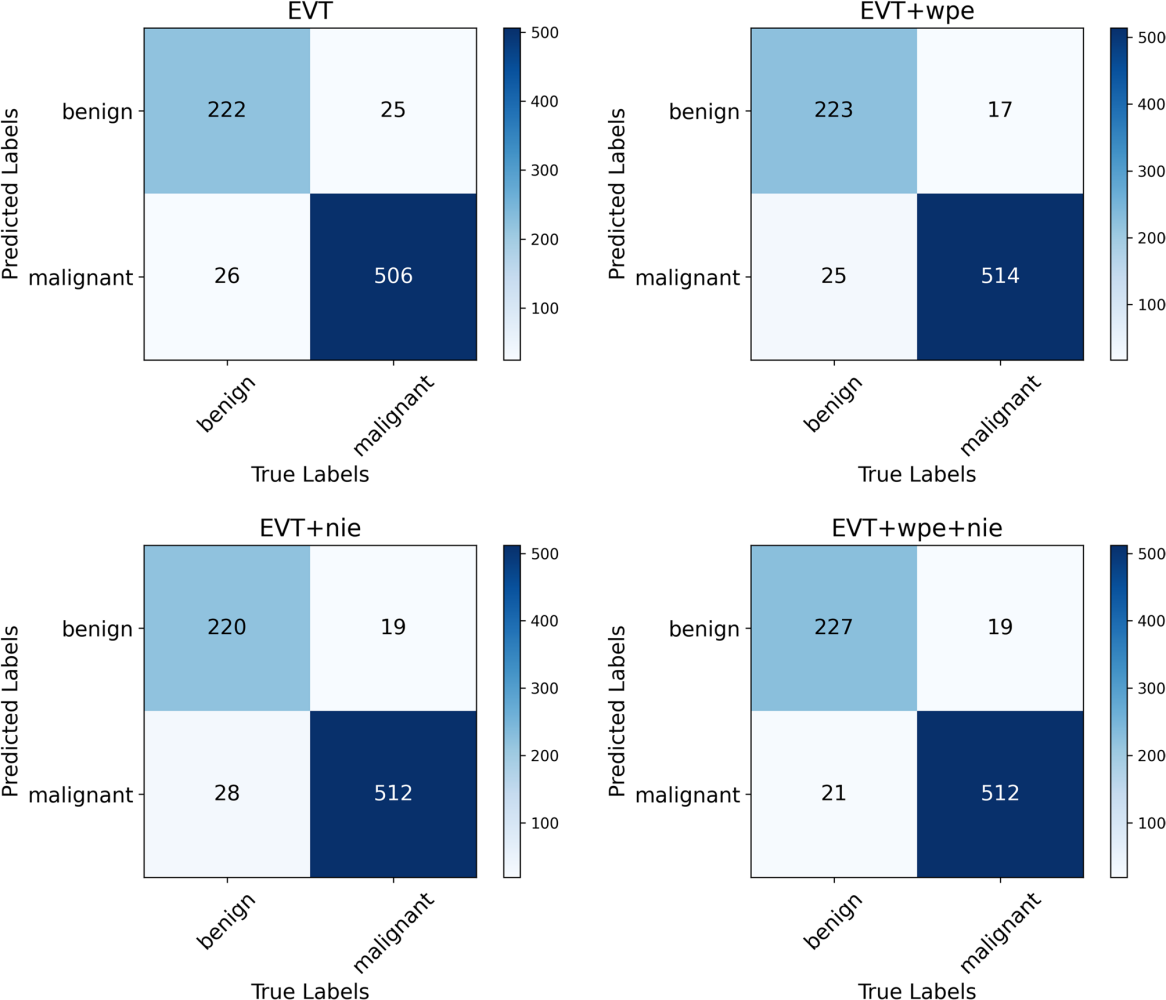
Confusion matrices comparing test data performance of EVT models with nuclear information enhancement and wavelet position transform.

**Fig. 7:**
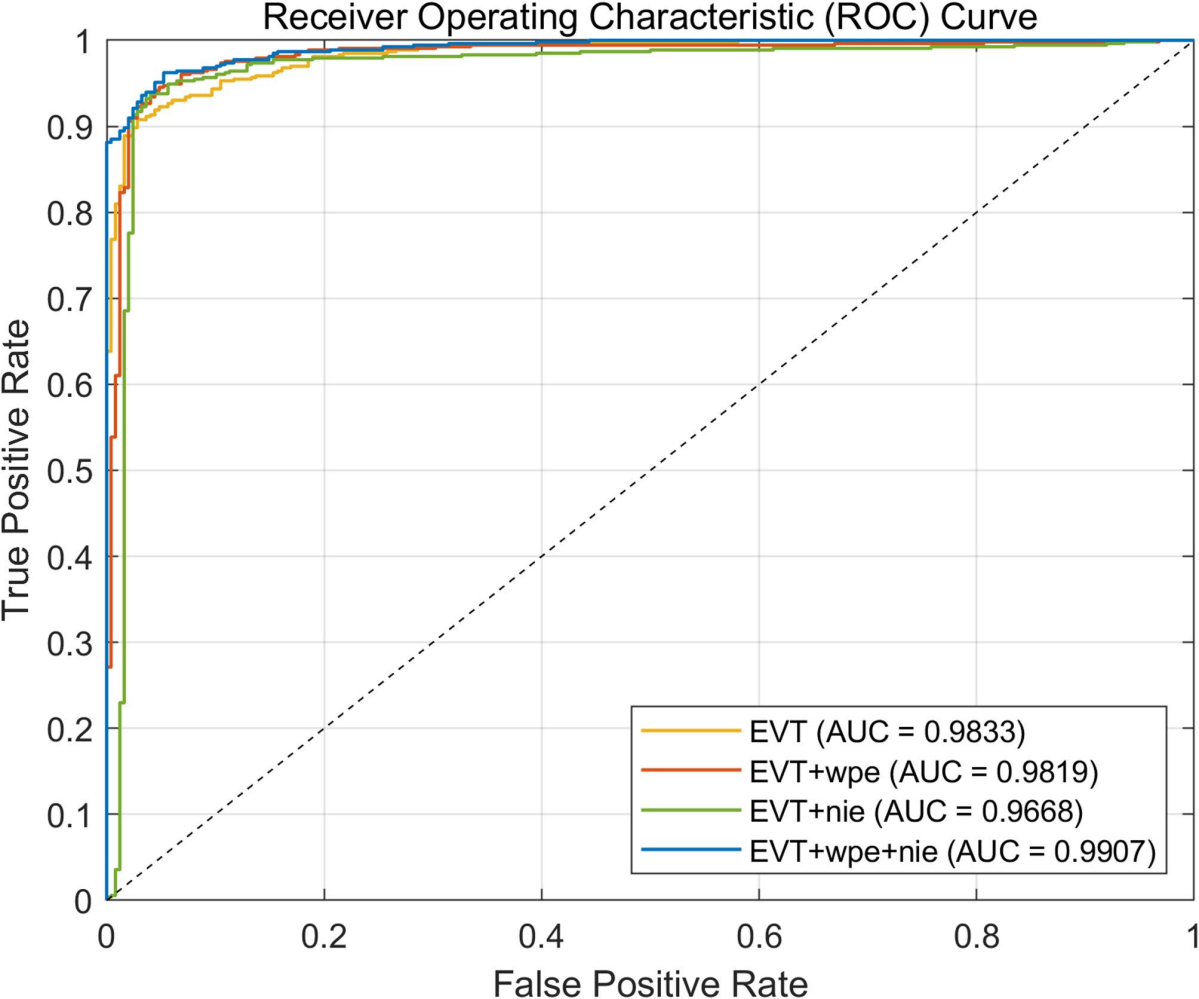
ROC curves and AUC values illustrating the classification performance of various EVT model combinations on test data.

The nie module focuses on enhancing the nuclear information features in the image, which enables the model to recognize and distinguish key nuclear structures in the image more accurately. The enhancement of the EVT + nie model is relatively small. However, when the nie module and the wpe module are jointly applied to the EVT model, the performance of the EVT + wpe + nie model is further improved, with an accuracy of 0.9487, a Youden index of 0.879, a Kappa value of 0.8814. We record the experimental of the confusion matrix, as shown in Fig. 6, and calculate the kappa value. The kappa value of the EVT + wpe + nie model has a significant increase, indicating a slight improvement in model consistency. We also plotted the ROC curves (Fig. 7), and the blue curve has the best classification cardinality up to 0.99, which is significantly higher than the other methods. This result further confirms the superiority of the synergistic effect of the two modules. The synergy of the two modules can mine image features more comprehensively, giving the EVT model a stronger classification capability when dealing with breast cancer pathology data exemplified by BreaKHis.

### Visual analysis

**Fig. 8:**
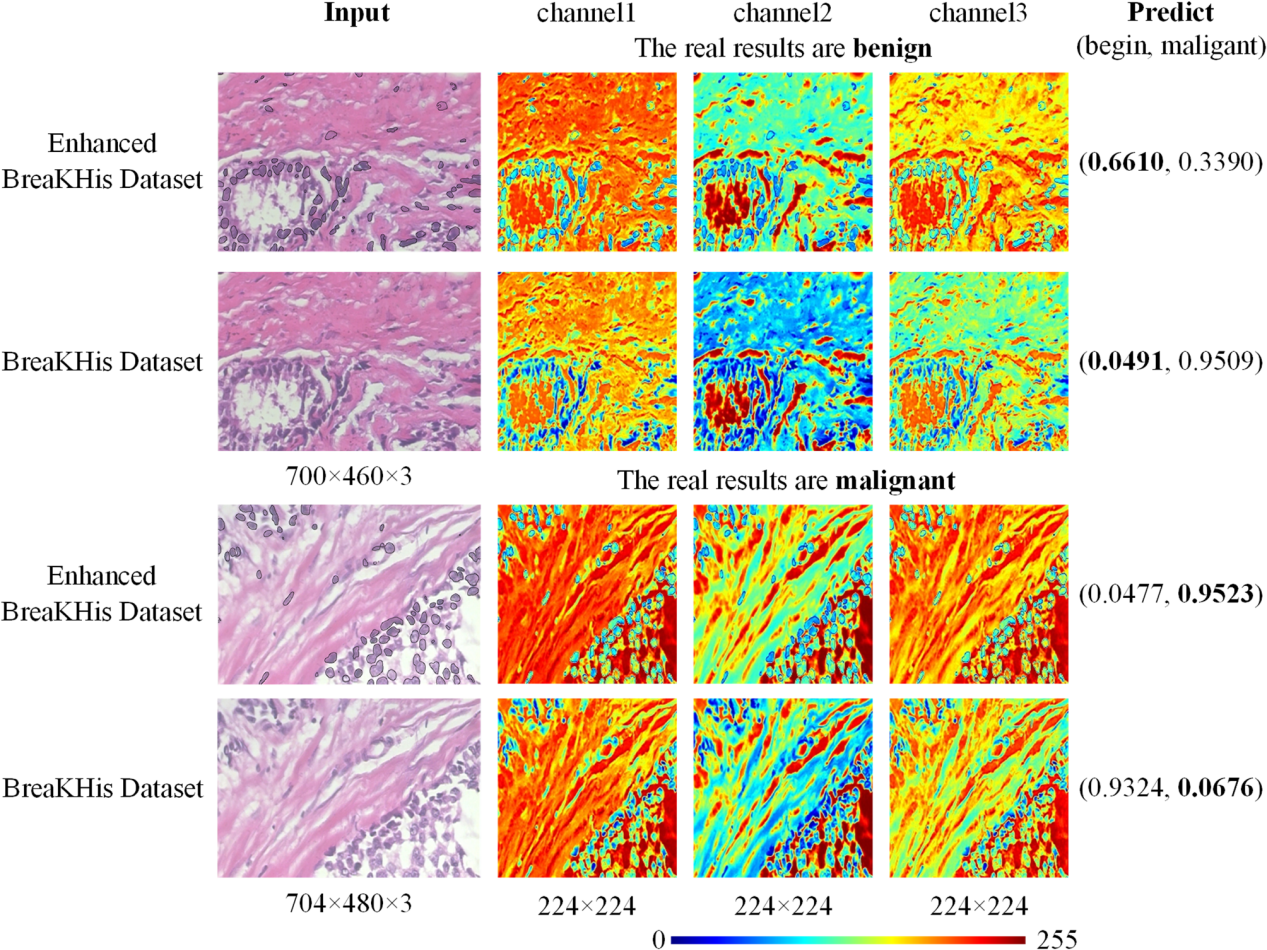
The color map is displayed after normalizing the tensor before the last flattener operation in the tokenizer.

To explore the specific effects of the nuclear enhancement method, we focused on the behavior of the tokenizer, a component network architecture, during data processing. We obtain the tensors before the tokenizer’s last flattener operation, which can be crucial for the subsequent Classifier. To visually demonstrate the key areas that a particular layer focuses on when processing data, we first carefully processed the tensor: First, we normalized each channel to ensure data consistency. Next, we scaled the normalized data by multiplying it by 255 to enhance visualization. Finally, we applied colormap technology to transform the processed tensor into an easily observable visualization, as shown in Fig. 8.

In two categories of begin and malignant, we selected images with incorrect prediction from the original data and images with correct prediction from the corresponding nuclear enhancement data for observation. We identified certain phenomena during network processing. Specifically, in the raw data, the tensor reflects a greater focus on the distribution of extracellular tissues across the three channels, while giving less weight to the nucleus, as shown by the blue areas representing the nucleus. This may hinder the network’s ability to recognize the nucleus and its related features, thereby reducing a classification accuracy. However, in the enhanced data, we found that the network not only continued to focus on the distribution of extracellular tissues, but also increased the weight given to the interior of the nucleus. This change allows the network to capture and identify the features of the nucleus, which is likely to be one of the factors contributing to the improved accuracy of classification. At the same time, we also noticed that a significant reduction in the network’s attention to the nuclear edge in the enhanced data. This suggests that the network may prioritize internal nuclear features over edge information due to the decreased weight assigned to the nuclear edge.

**Fig. 9:**
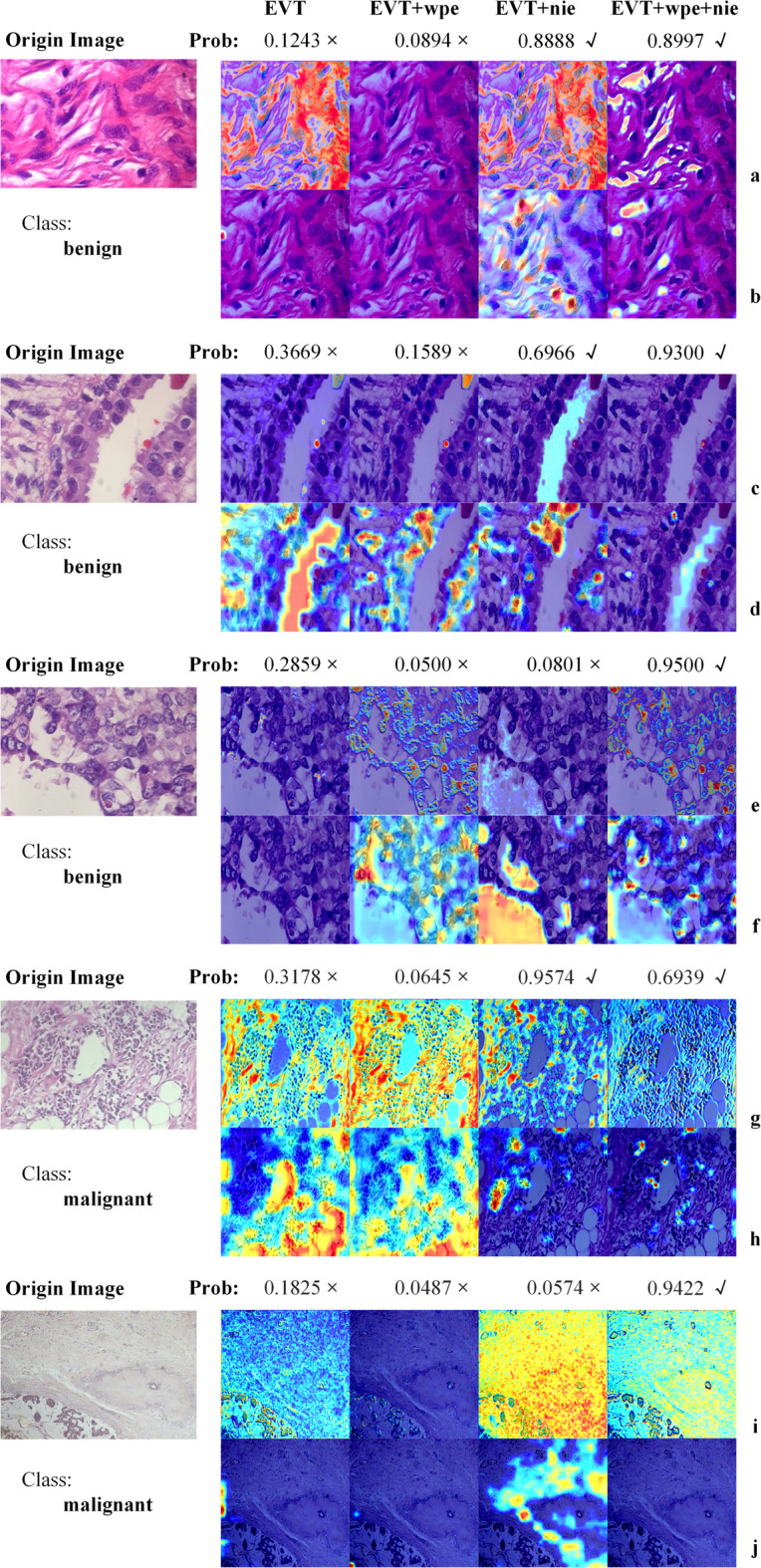
Network model visualization. The a, c, e, g and i is the *model.tokenizer.conv_layers[0][0]* layer, the first convolutional layer in the network. The b, d, f, h and j is the *Model.Tokenizer.conv_layers[2][2]* layer, which is the last maximum pooling layer of the tokenizer module.

To better understand the role of cytosolic enhancement in EVT, we further used Grad-CAM (Gradient-weighted Class ActivationMapping) technique^52^ to visualize the key layers of the network. With Grad-CAM, we can visualize how much the network pays attention to different regions in the decision-making process, and thus observe the effect of nucleus enhancement on the network^48^. The specific visualization results are shown in Fig. 9. We chose two layers, *model.tokenizer.conv_layers[0][0]*(Fig. 9acegi) and *model.tokenizer.conv_layers[2][2]*(Fig. 9bdfhj), for visualization. The *model.tokenizer.conv_layers[0][0]* is the first convolutional layer in the network, which is responsible for extracting low-level features of the input data, such as edges and texture information, which are crucial for subsequent feature extraction and classification tasks. And the *model.tokenizer.conv_layers[2][2]* is the last maximum pooling layer in the tokenizer module, which serves to reduce the spatial dimensionality of the feature maps while retaining the key feature information, thus reducing the computational effort and enhancing the robustness of the features. The visualization results of this layer help to reveal the information processing and filtering mechanisms of the network before and after data enhancement at the end stage of feature extraction.

In Fig. 9, by comparing EVT and EVT + nie network visualizations in subfigures b, c, d, e, g and h, it is evident that nuclear enhancement leads to a significant reduction in the weight of surrounding tissue. However, in subfigures f, i, and j, despite the application of data enhancement, the model failed to properly reduce the weight of the surrounding tissue, resulting in a decline in the accuracy of the predicted results. This phenomenon further confirms the necessity of appropriately reducing the weight of surrounding tissue to improve the accuracy of prediction in image analysis. When we combine nuclear information (nie) enhancement with wavelet position transformation (wpe) technology, as shown in subfigures e and g comparing EVT + wpe and EVT + wpe + nie network visualization, we can see that this combination can capture nuclear edge features more accurately. Further analysis of the results of EVT + nie and EVT + wpe + nie in subfigures e, f, i and j shows that, in some cases, when the model misunderstands some features, the nuclear boundary features can be obtained more clearly after combining the wavelet position transformation, and the weight of the surrounding tissue is relatively weakened, and the inference can be correct.

Convolutional Neural Networks (CNNs)​​ primarily rely on ​​local receptive fields​​ (small kernel convolutions), inherently probably limiting their capacity to model ​​​in histopathology images (citation needed)^29^. It can be known from the Grad-CAM visualization (Fig. 9) that the EVT_wpe model based on NIE significantly reduces the attention to non-kernel regions (such as interstitium) in the decision-making process. As demonstrated in ​​Table 4, a CNN (e.g., ResNet34) may misclassify benign regions with dense stromal texture as malignant (recall: 0.898) due to its inability to disambiguate local textural similarities, whereas the ​​EVT_wpe​​ model achieves superior recall (0.968) by leveraging global context. In summary, the results of this study show that the combination of nuclear enhancement and wavelet position transformation technology can effectively improve the localization of nuclear edge in image analysis and reduce the weight of extranuclear tissue, which has important theoretical significance for improving the accuracy of pathological image classification.

## Conclusions

In this paper, we propose a segmentation model-based classification and processing framework for breast cancer pathological images, aimed at enhancing the accuracy of classification through data enhancement techniques. The introduced data processing framework, nuclear enhancement combined with wavelet position transformation, can more accurately locate nuclear boundaries in image visualization analysis, further weaken the weight of extranuclear tissue information, and thus optimize the pertinence and accuracy of feature extraction. This has a favorable influence on the model’s comprehensive analysis of the pathological image. Furthermore, we introduce an EVT utilizing wavelet position embedding and demonstrate its feasibility and effectiveness by comparing it with the baseline network structure on the BreaKHis dataset.

Currently, the high-performance networks are extensively utilized in medical image analysis, and our EVT, grounded in wavelet position embedding, offers robust support for breast cancer pathological image classification research and exhibits promising performance. However, it is important to acknowledge that our study still has limitations. For instance, the segmentation model used for nucleus extraction has considerable potential for improvement, and the classification performance may be further enhanced with more accurate segmentation. In the future, more advanced hyperparameter optimization techniques will be explored. In response to the criticism of our framework, the performance of the EVT model can be further improved by adjusting the intensity of nuclear information enhancement. It is noteworthy that, despite the nucleus segmentation model selected in this study having considerable potential for improvement, the classification effect still demonstrates a positive effect, even when the nucleus segmentation quality is suboptimal. Hence, it can be inferred that as the segmentation performance improves, the corresponding classification performance will also elevate. Breast cancer typically arises from intraductal carcinoma, where cancerous cells breach the ductal basement membrane and invade the stromal cells. To further improve tumor screening accuracy, future work could explore catheter-stromal interactions in pathological images. Additionally, the generalizability of our method should be validated across diverse datasets to ensure robust clinical performance.

## Data Availability

The implementation details and associated code for this study are publicly available on GitHub: https://github.com/Zichuana/Enhanced-Nuclear-Information-Fusion-and-Visual-Transformer. The GitHub repository is organized into main folders for data and code, with data divided into masks and processed subfolders and code categorized by visualization and network structure scripts. The datasets used and analysed during the current study available from the corresponding author on reasonable request. This includes the SNOW dataset derived from https://zenodo.org/records/6633721, the TNBC dataset from https://zenodo.org/records/1175282#.YMisCTZKgow, and the BreaKHis dataset through https://www.kaggle.com/datasets/anaselmasry/breast-cancer-dataset for datasets.

## References

[1] Sung, H., Ferlay, J. & Siegel, R. L., et al. Global cancer statistics 2020: GLOBOCAN estimates of incidence and mortality worldwide for 36 cancers in 185 countries. *CA* **71**(3), 209–249 (2021). 10.3322/caac.21660 33538338

[2] Gomes, D. S. et al. Inter-observer variability between general pathologists and a specialist in breast pathology in the diagnosis of lobular neoplasia, columnar cell lesions, atypical ductal hyperplasia and ductal carcinoma in situ of the breast. *Diagn. Pathol.* **9**, 1–9 (2014). 24948027 10.1186/1746-1596-9-121 PMC4071798

[3] Kaul, V., Enslin, S. & Gross, S. A. History of artificial intelligence in medicine. *Gastrointest. Endosc.* **92**(4), 807–812 (2020). 32565184 10.1016/j.gie.2020.06.040

[4] LeCun, Y., Bengio, Y. & Hinton, G. Deep learning. *Nature* **521**(7553), 436–444 (2015). 26017442 10.1038/nature14539

[5] Ibrahim, A. et al. Artificial intelligence in digital breast pathology: Techniques and applications. *The Breast* **49**, 267–273 (2020). 31935669 10.1016/j.breast.2019.12.007 PMC7375550

[6] Buch, V. H., Ahmed, I. & Maruthappu, M. Artificial intelligence in medicine: Current trends and future possibilities. *Br. J. Gen. Pract.* **68**(668), 143–144 (2018). 29472224 10.3399/bjgp18X695213 PMC5819974

[7] Jabeen, K., Khan, M. A. & Hamza, A., et al. An EfficientNet integrated ResNet deep network and explainable AI for breast lesion classification from ultrasound images. *CAAI Trans. Intell. Technol*. (2024).

[8] Dosovitskiy, A. An image is worth 16x16 words: Transformers for image recognition at scale. arXiv preprint arXiv:2010.11929, 2020.

[9] Chen, H. et al. GasHis-Transformer: A multi-scale visual transformer approach for gastric histopathological image detection. *Pattern Recognit.* **130**, 108827 (2022).

[10] Zou, X. et al. Delving deeper into anti-aliasing in convnets. *Int. J. Comput. Vis.* **131**(1), 67–81 (2023).

[11] Ding, M. et al. An enhanced vision transformer with wavelet position embedding for histopathological image classification. *Pattern Recognit.* **140**, 109532 (2023).

[12] Guyon, I. & Elisseeff, A. An Introduction to Feature Extraction. Feature Extraction: Foundations and Applications. Berlin, Heidelberg: Springer Berlin Heidelberg (2006), 1–25.

[13] Naylor, P. et al. Segmentation of nuclei in histopathology images by deep regression of the distance map. *IEEE Trans. Med. Imaging* **38**(2), 448–459 (2018). 10.1109/TMI.2018.2865709 30716022

[14] Elmore, J. G. et al. Screening for breast cancer. *JAMA* **293**(10), 1245–1256 (2005). 15755947 10.1001/jama.293.10.1245 PMC3149836

[15] Pienta, K. J., Partin, A. W. & Coffey, D. S. Cancer as a disease of DNA organization and dynamic cell structure. *Cancer Res.* **49**(10), 2525–2532 (1989). 2653618

[16] Pienta, K. J. & Coffey, D. S. Correlation of nuclear morphometry with progression of breast cancer. *Cancer* **68**(9), 2012–2016 (1991). 1655233 10.1002/1097-0142(19911101)68:9<2012::aid-cncr2820680928>3.0.co;2-c

[17] Wu, M. & Ma, J. Association between imaging characteristics and different molecular subtypes of breast cancer. *Acad. Radiol.* **24**(4), 426–434 (2017). 27955963 10.1016/j.acra.2016.11.012

[18] Sabottke, C. F. & Spieler, B. M. The effect of image resolution on deep learning in radiography. *Radiol. Artif. Intell.* **2**(1), 90015 (2020). 10.1148/ryai.2019190015 PMC8017385 33937810

[19] Koziarski, M. & Cyganek, B. Impact of low resolution on image recognition with deep neural networks: An experimental study. *Int. J. Appl. Math. Comput. Sci.* **28**(4), 735–744 (2018).

[20] Srinidhi, C. L., Ciga, O. & Martel, A. L. Deep neural network models for computational histopathology: A survey. *Med. Image Anal.* **67**, 101813 (2021). 33049577 10.1016/j.media.2020.101813 PMC7725956

[21] Fatima, M., Khan, M, A. & Shaheen, S., et al. Breast lesion segmentation and classification using U-Net saliency estimation and explainable residual convolutional neural network. *Fractals* **10**, S0218348X24400607 (2024).

[22] Ortega-Ruíz, M. A., Karabağ, C. & Roman-Rangel, E., et al. DRD-UNet, a UNet-like architecture for multi-class breast cancer semantic segmentation. *IEEE Access* (2024).

[23] Ding, K. et al. A large-scale synthetic pathological dataset for deep learning-enabled segmentation of breast cancer. *Sci. Data* **10**(1), 231 (2023). 37085533 10.1038/s41597-023-02125-y PMC10121551

[24] Spanhol, F. A. et al. A dataset for breast cancer histopathological image classification. *IEEE Trans. Biomed. Eng.* **63**(7), 1455–1462 (2015). 26540668 10.1109/TBME.2015.2496264

[25] Seo, H., Brand, L. & Barco, L. S., et al. Scaling multi-instance support vector machine to breast cancer detection on the BreaKHis dataset. *Bioinformatics* **38**(Supplement_1): i92–i100 (2022). 10.1093/bioinformatics/btac267 PMC9235475 35758811

[26] Xiao, M. X., Li, Y. & Yan, X., et al. Convolutional neural network classification of cancer cytopathology images: Taking breast cancer as an example. *Proceedings of the 2024 7th International Conference on Machine Vision and Applications*, 145–149 (2024).

[27] Simonyan, E. O., Badejo, J. A. & Weijin, J. S. Histopathological breast cancer classification using CNN. *Mater. Today Proc.* **105**, 268–275 (2024).

[28] Rosales-Morales, A. E., Gutiérrez-Alfaro, A. & Ornelas-Rodríguez, M., et al. Generative models for class imbalance problem on breakhis dataset: A case study. New Horizons for Fuzzy Logic, Neural Networks and Metaheuristics. Cham: Springer Nature Switzerland, 105–119 (2024).

[29] Vaswani, A. Attention is all you need. Advances in Neural Information Processing Systems (2017).

[30] Atabansi, C. C. et al. A survey of Transformer applications for histopathological image analysis: New developments and future directions. *Biomed. Eng. Online* **22**(1), 96 (2023). 37749595 10.1186/s12938-023-01157-0 PMC10518923

[31] Veta, M. et al. Breast cancer histopathology image analysis: A review. *IEEE Trans. Biomed. Eng.* **61**(5), 1400–1411 (2014). 24759275 10.1109/TBME.2014.2303852

[32] Xiao, H. et al. Transformers in medical image segmentation: A review. *Biomed. Signal Process. Control* **84**, 104791 (2023).

[33] Shamshad, F. et al. Transformers in medical imaging: A survey. *Med. Image Anal.* **88**, 102802 (2023). 37315483 10.1016/j.media.2023.102802

[34] Saleem, S. & Sharif, M. I. An integrated deep learning framework leveraging NASNet and vision transformer with mixprocessing for accurate and precise diagnosis of lung diseases. arXiv preprint arXiv:2502.20570, 2025. 10.1016/j.slast.2026.100394 41580084

[35] Shou, Y. et al. Object detection in medical images based on hierarchical transformer and mask mechanism. *Comput. Intell. Neurosci.* **2022**(1), 5863782 (2022). 35965770 10.1155/2022/5863782 PMC9371842

[36] Krithiga, R. & Geetha, P. Breast cancer detection, segmentation and classification on histopathology images analysis: A systematic review. *Arch. Comput. Methods Eng.* **28**(4), 2607–2619 (2021).

[37] Yu, X. et al. Unest: local spatial representation learning with hierarchical transformer for efficient medical segmentation. *Med. Image Anal.* **90**, 102939 (2023). 37725868 10.1016/j.media.2023.102939 PMC11229077

[38] Riasatian, A. et al. Fine-tuning and training of densenet for histopathology image representation using tcga diagnostic slides. *Med. Image Anal.* **70**, 102032 (2021). 33773296 10.1016/j.media.2021.102032

[39] Wang, F. et al. Multi-granularity cross-modal alignment for generalized medical visual representation learning. *Adv. Neural. Inf. Process. Syst.* **35**, 33536–33549 (2022).

[40] Maleki, D. & Tizhoosh, H. R. LILE: Look in-depth before looking elsewhere–a dual attention network using transformers for cross-modal information retrieval in histopathology archives. *International Conference on Medical Imaging with Deep Learning. PMLR*, 879–894 (2022).

[41] Raminedi, S., Shridevi, S. & Won, D. Multi-modal transformer architecture for medical image analysis and automated report generation. *Sci. Rep.* **14**(1), 19281 (2024). 39164302 10.1038/s41598-024-69981-5 PMC11336090

[42] Li, W. et al. High resolution histopathology image generation and segmentation through adversarial training. *Med. Image Anal.* **75**, 102251 (2022). 34814059 10.1016/j.media.2021.102251

[43] Wang, Z. & Sun, J. Survtrace: Transformers for survival analysis with competing events. *Proceedings of the 13th ACM International Conference on Bioinformatics, Computational Biology and Health Informatics.,* 1–9 (2022).

[44] Wetstein, S. C. et al. Deep learning-based breast cancer grading and survival analysis on whole-slide histopathology images. *Sci. Rep.* **12**(1), 15102 (2022). 36068311 10.1038/s41598-022-19112-9 PMC9448798

[45] Khader, F. et al. Medical transformer for multimodal survival prediction in intensive care: Integration of imaging and non-imaging data. *Sci. Rep.* **13**(1), 10666 (2023). 37393383 10.1038/s41598-023-37835-1 PMC10314902

[46] Zhu, X., Yao, J. & Zhu, F., et al. Wsisa: Making survival prediction from whole slide histopathological images. *Proceedings of the IEEE Conference on Computer Vision and Pattern Recognition*. 7234–7242 (2017).

[47] Sriwastawa, A. & Arul Jothi, J. A. Vision transformer and its variants for image classification in digital breast cancer histopathology: a comparative study.* Multimed. Tools Appl*. **83**, 39731–39753 (2024).

[48] Springenberg, M., Frommholz, A. & Wenzel, M., et al. From cnns to vision transformers—A comprehensive evaluation of deep learning models for histopathology. arXiv preprint arXiv:2204.05044 (2022). 10.1016/j.media.2023.102809 37201221

[49] Qian, S. et al. Blending anti-aliasing into vision transformer. *Adv. Neural. Inf. Process. Syst.* **34**, 5416–5429 (2021).

[50] Fischer, E. G. Nuclear morphology and the biology of cancer cells. *Acta Cytol.* **64**(6), 511–519 (2020). 32570234 10.1159/000508780

[51] Bhalla, K. et al. Radiologic imaging biomarkers in triple-negative breast cancer: a literature review about the role of artificial intelligence and the way forward. *BJR Artif. Intell.* **1**(1), ubae016 (2024). 40201726 10.1093/bjrai/ubae016 PMC11974408

[52] Selvaraju, R. R. et al. Grad-CAM: visual explanations from deep networks via gradient-based localization. *Int. J. Comput. Vis.* **128**, 336–359 (2020).

